# Supplementary material for: Deletion of Glycogen Synthase Kinase 3 Beta Reprograms NK Cell Metabolism
Source: Cancers (Basel). 2023 Jan 24;15(3):705. doi: 10.3390/cancers15030705 (PMC9913837; doi:10.3390/cancers15030705)
Supplement: Supplementary file 1 [file cancers-15-00705-s001.zip › cancers-2171103-supplementary.pdf]

Supplementary figures.

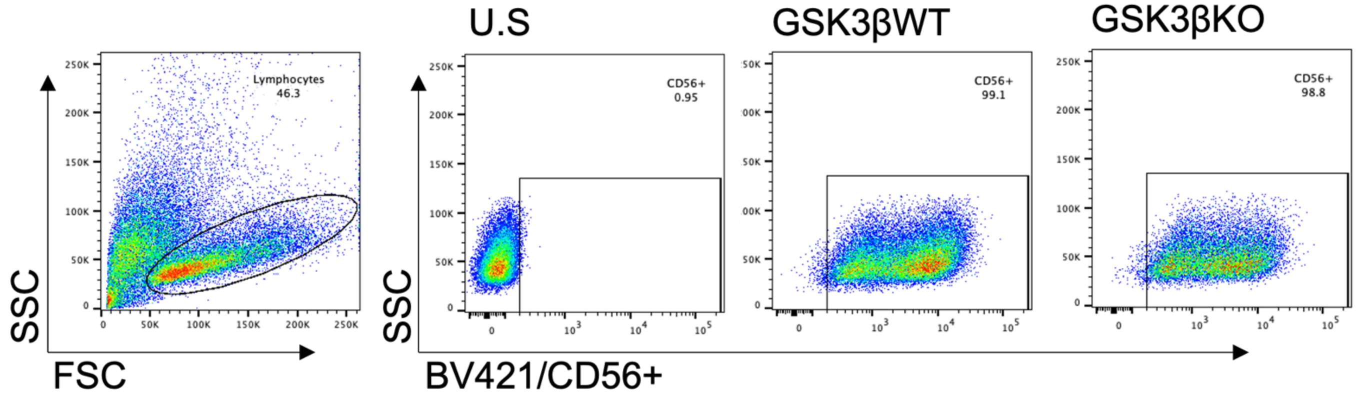

Figure S1. Flow cytometry gating strategy to evaluate CD56 expression on NK cells. Purified and expanded NK cells were gated based on differential morphology FSC vs SSC - lymphocyte gating strategy. NK cells were evaluated for BV421/CD56+ cells right after lymphocyte gating strategy. Data suggests that deletion of GSK3B did not alter CD56 expression on NK cells.

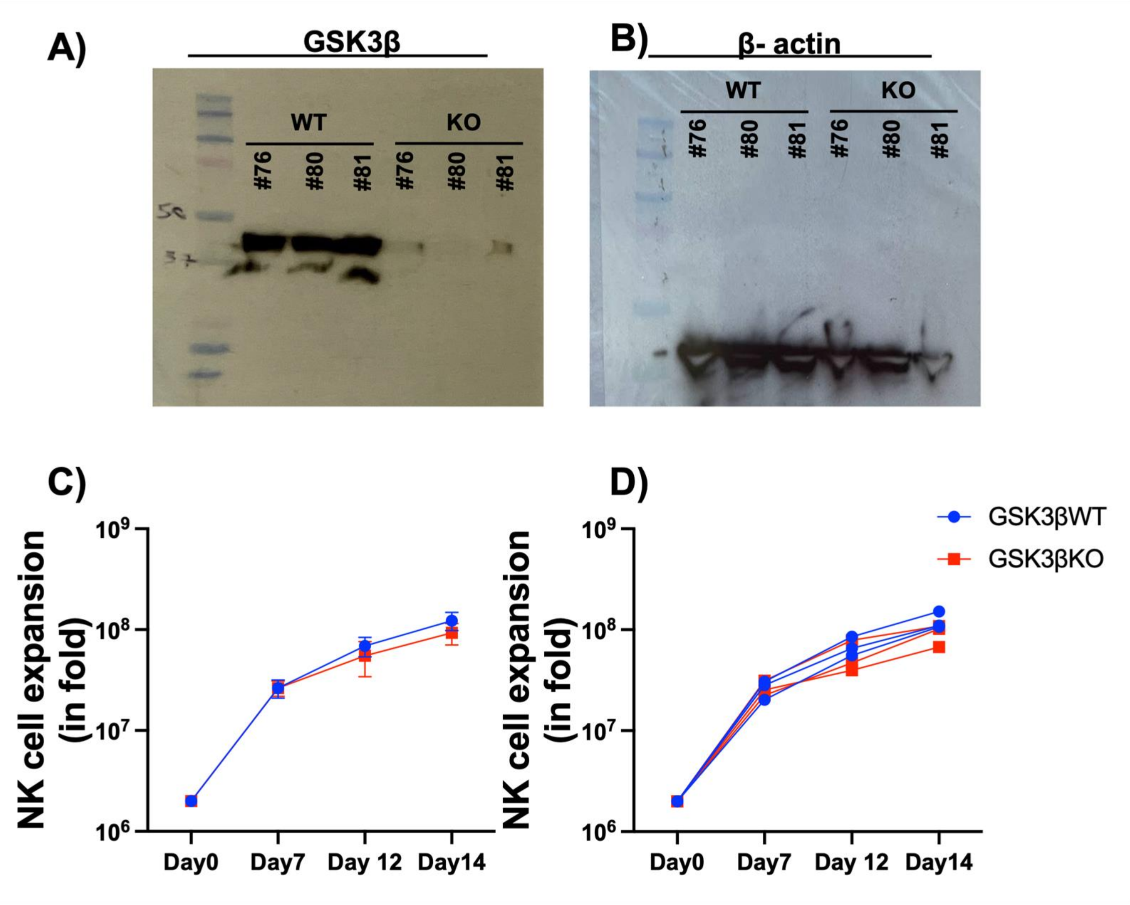

Figure S2. Protein expression on NK cells after CRISPR/Casp9. Generation of *GSK3B-KO* and CRISPR efficiency was evaluated by Western Blot (A and B). Cell proliferation after *GSK3B* deletion on NK cells. (n=3, C shows data from three donors and. D shows data from individual donors).

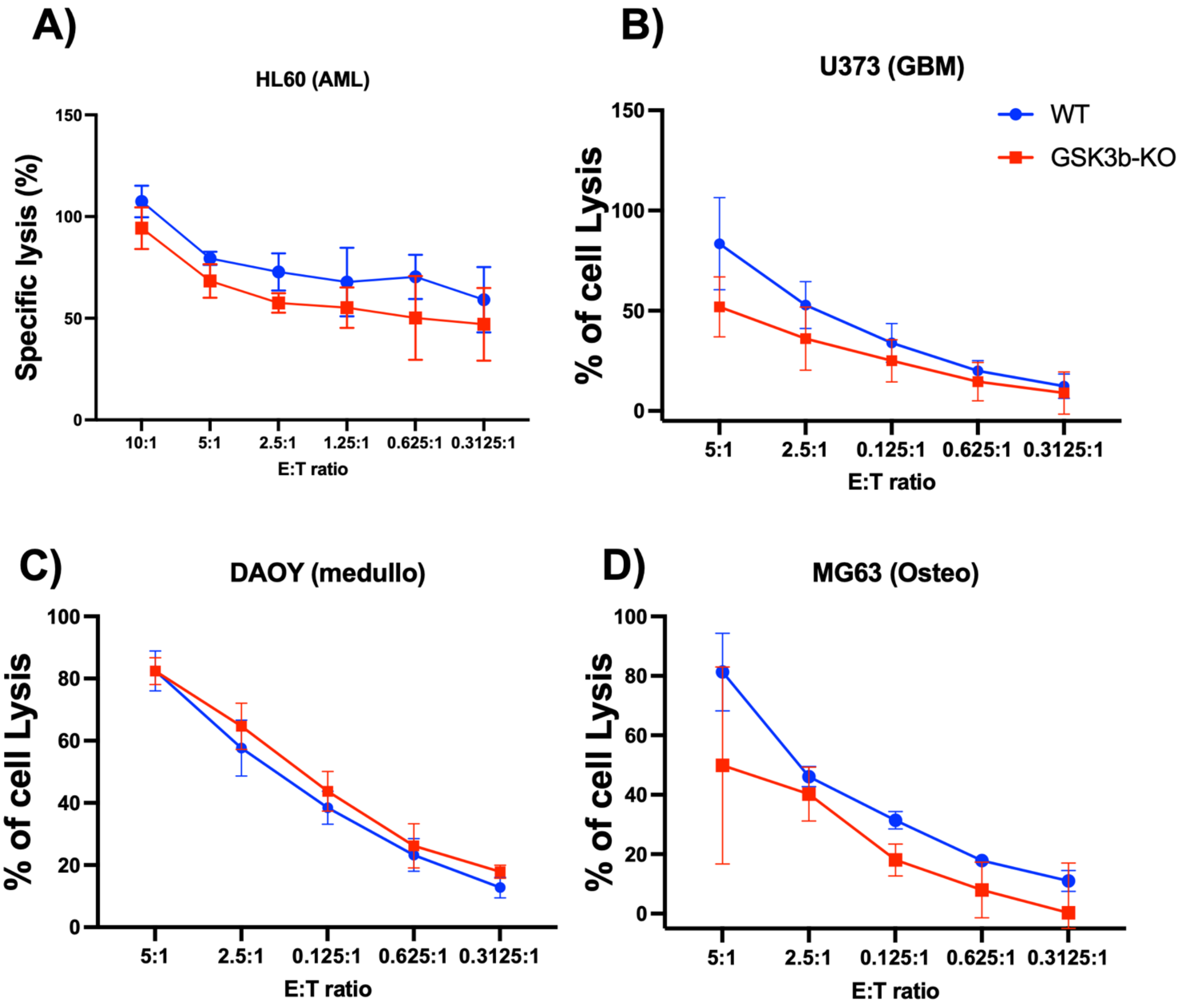

Figure S3. WT and *GSK3B*-KO NK cells showed similar killing potency. Both WT and *GSK3B*-KO NK cells were cocultured with HL60 (AML) (A), U373 (Glioblastoma) (B), DAOY (medulloblastoma), and MG63 (osteosarcoma). NK cell killing is shown as percentage of cell lysis (n=3).

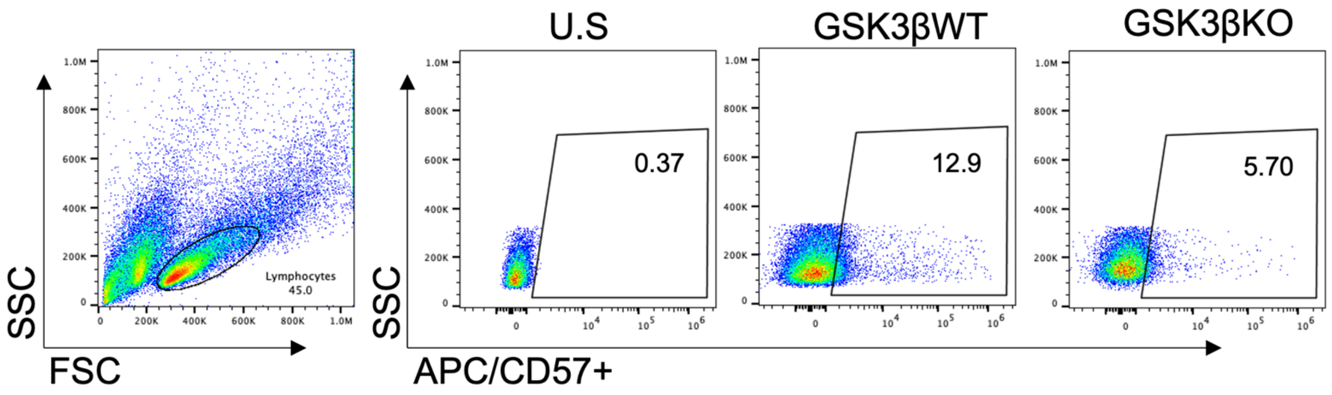

Figure S4. Flow cytometry gating strategy to evaluate CD57⁺ cells. Purified NK cells were gated based on differential morphology FSC vs SSC - lymphocyte gating strategy. NK cells were evaluated for APC/CD57+ cells right after lymphocyte gating strategy.
